# A Novel Regulator of Preadipocyte Differentiation, Transcription Factor TCF21, Functions Partially Through Promoting LPL Expression

**DOI:** 10.3389/fphys.2019.00458

**Published:** 2019-04-23

**Authors:** Xinyang Zhang, Bohan Cheng, Chang Liu, Zhiqiang Du, Hui Zhang, Ning Wang, Mengqi Wu, Yumao Li, Zhiping Cao, Hui Li

**Affiliations:** ^1^Key Laboratory of Chicken Genetics and Breeding, Ministry of Agriculture and Rural Affairs, Harbin, China; ^2^Key Laboratory of Animal Genetics, Breeding and Reproduction, Education Department of Heilongjiang Province, Harbin, China; ^3^College of Animal Science and Technology, Northeast Agricultural University, Harbin, China

**Keywords:** TCF21, preadipocyte, differentiation, chicken, LPL

## Abstract

The transcription factor TCF21 has been previously shown to be specifically expressed in white preadipocytes in mice. However, the exact biological function of TCF21 in the context of adipogenesis remains unknown. In the current study, we used chicken lines selected based on their abdominal fat content, and observed a significant decrease in TCF21 mRNA and protein levels in the abdominal fat of lean broilers relative to fat broilers. Moreover, TCF21 expression increased throughout preadipocyte differentiation *in vitro*. We also found that TCF21 knockdown and over-expression attenuated and promoted preadipocyte differentiation, respectively, as evidenced by appropriate changes in lipid droplet accumulation and altered expressions of *C/EBPa*, *LPL*, and *A-FABP*. Additional chromatin immunoprecipitation analyses and luciferase assays demonstrated that TCF21 promotes the transcription of *LPL* by directly binding to the E-box motif in the *LPL* promoter. Together, these results show that TCF21 is a novel regulator of preadipocyte differentiation, in part by directly promoting LPL expression.

## Introduction

Transcription factor 21 belongs to the bHLH protein family, characterized by a HLH domain which mediates protein interactions and a highly charged basic region which mediates DNA binding site recognition (Jones, 2004). TCF21 plays important roles in the development of a variety of tissues, including the heart, lung, spleen, kidney, and gonads (Tandon et al., 2013). However, despite several recent reports, the role of TCF21 in the growth and development of adipose tissue remain poorly understood. Microarray analyses of brown and white preadipocytes revealed that TCF21 is not expressed in brown preadipocytes, and is instead specific to white preadipocytes, which suggests that TCF21 may be useful as a biomarker of white preadipocytes (Timmoms et al., 2007). Several subsequent studies in human and mice have shown that the abundance of TCF21 mRNA decreases as cells transition from white to brown adipocytes (Petrovic et al., 2010; Elsen et al., 2014; Guennoun et al., 2015). In addition, TCF21 expression is significantly higher in the visceral adipose tissue of obese Uygurs (Zhang et al., 2015) and obese mice (Stadion et al., 2018) compared to their normal weight counterparts. Therefore, TCF21 may play an important role in regulating white adipose tissue development.

To our knowledge, no prior studies have examined the effect of TCF21 on fat deposition in chickens. Using broilers divergently selected for their strikingly different abdominal fat content, a prior study showed that TCF21 expression was significantly associated with TeW and testis ratio (TeW/body weight) in male broilers (Zhang et al., 2017), reflecting the well-known link between fat levels and reproductive abilities. Given prior findings regarding the role of TCF21 in adipogenesis in mice and humans, we hypothesize that in addition to its effects on testis development, TCF21 may also be vital to the regulation of chicken adipose tissue growth and development. As such, this study aimed to explore the function of TCF21 during chicken preadipocyte differentiation and the molecular mechanisms governing this function.

## Materials and Methods

### Ethics Statement

All animal studies performed in this publication were approved by the Laboratory Animal Management Committee of the Northeast Agricultural University (Harbin, China) and were performed in accordance with the guidelines produced by the Ministry of Science and Technology of the People’s Republic of China (Approval No. 2006-398) regarding the care and use of experimental animals.

The research project was approved by the Institutional Biosafety Committee of Northeast Agriculture University (Harbin, China). Plasmid construction and transfection were performed in accordance with the guidelines for the Regulation on Safety Administration of Agricultural Genetically Modified Organisms (RSAGMO) established by the People’s Republic of China (Revised version 2017). All commonly required, special biosecurity measures were undertaken in the laboratory during experiments.

### Animals

A total of 70 male chickens (35 each from lean and fat lines) from the 19^th^ generation (G_19_) population of the NEAUHLF were used. The selection procedure of NEAUHLF has been described in detail previously (Guo et al., 2011). In G_19_, the abdominal fat percentage [AFP = abdominal fat weight (AFW)/body weight at 7 weeks of age (BW7)] of fat birds was 6.8 times that of lean birds. All animals were housed in the same environment with free access to food and water. Birds were given starter feed from birth to 3 weeks of age (3,100 kcal of ME/kg; 210 g/kg of CP), and then switched to a grower diet from 4 to 7 weeks of age (3,000 kcal of ME/kg; 190 g/kg of CP). Each week from weeks 1 to 7, 5 birds from each of the two lines were sacrificed after a 10 h fasting period.

### Tissue Collection

Following sacrifice, abdominal fat was collected from each bird, washed with 0.75% NaCl, snap frozen with liquid nitrogen, and transferred to -80°C for storage prior to use.

### Cell Fraction Preparation and Preadipocyte Culture Conditions

Chicken stromal-vascular and fat cells were isolated from the abdominal fat tissue of 10-day-old AA broilers according to a previously published protocol (Zhang et al., 2014; Cheng et al., 2016). Isolated preadipocytes (stromal-vascular cells) were plated at 1 × 10^5^ cells/ cm^2^ in DMEM media (Gibco, New York, NY, United States) containing 10% FBS (Biological Industries), and grown in a standard humidified incubator. Once cells achieved > 90% confluency, they were passaged and plated at 1 × 10^5^ cells/ cm^2^ in 6-well plates. Once these plated cells achieved 50% confluency, differentiation media was generated by adding 160 μM oleate to the existing media, this induced preadipocyte differentiation. Media was exchanged daily through day 5 of the differentiation process.

### Oil Red-O Staining

Adipocyte lipid accumulation was assessed via oil red-O staining. First, cells were washed three times in PBS, and then fixed in 4% paraformaldehyde for 30 min at room temperature. Cells were again washed using PBS, and then stained with freshly diluted oil red-O (oil red-O stock solution: distilled H_2_O = 3:2) for 15 min. Lipid droplets were imaged using an inverted fluorescent microscope (Leica). Next, excess stain was removed using a small transfer pipette, and cells were washed five to six times with distilled water. Oil red-O was extracted from the cells using a 100% propan-2-ol solution, and absorbance was measured at 510 nm. Absorbance readings were then normalized to the cell number input in order to generate accurate comparisons.

### Quantitative Polymerase Chain Reaction

RNA was extracted using TRIzol Reagent (Invitrogen, Carlsbad, CA, United States) according to manufacturer’s instructions. Collected RNA was diluted using nuclease-free water and electrophoresed on a denaturing formaldehyde agarose gel to visualize rRNA and ensure overall sample quality. Samples that had a 28S:18S ratio of 1.8–2.1 were deemed good quality and used for follow-up experiments.

Reverse transcription was performed on 1 μg total RNA from each sample using the ImProm-II^TM^ Reverse Transcription System (Promega, Madison, WI, United States) according to manufacturer’s instructions, and a 7500 Real-time PCR System (Applied Biosystems, Foster City, CA, United States) was utilized to conduct the real time quantitative PCR (qPCR) reactions. FastStart Universal SYBR Green Master Mix (Roche, Indianapolis, IN, United States) was used for qPCR reactions, using 1 μL cDNA, and appropriate volumes of specific primers in a final 10 μL volume. qPCR cycling conditions were as follows: 95°C for 10 min; 40 cycles of 95°C for 15 s then 60°C for 1 min. Triplicate reactions were performed to ensure accuracy. Gene expression was normalized to that of TATA-box binding protein (*TBP*), and the 2^-Δ^ ^CT^ method was used for expression calculations (Schmittgen and Livak, 2008) [ΔCT = CT (target gene) – CT (*TBP*)]. Primer sequences are given in Table 1.

**Table 1:** qPCR primer sequences.

Gene	Accession number	Primer sequence (5′ to 3′)
*TCF21*	NM_001277711	F: ACGCTGCCAACGCAAGGG
		R: TGTTCACCACTTCTTTCAGGTCACTC
*PPARγ*	NM_001001460	F: GTGCAATCAAAATGGAGCC
		R: CTTACAACCTTCACATGCAT
*C/EBPa*	NM_001031459	F: GCGACATCTGCGAGAACG
		R: GTACAGCGGGTCGAGCTT
*A-FABP*	NM_204290	F: ATGTGCGACCAGTTTGT
		R: TCACCATTGATGCTGATAG
*LPL*	NM_205282	F: ATGTTCATTGATTGGATGGAGGAG
		R: AAAGGTGGGACCAGCAGGAT
*PLIN*	NM_001127439	F: GGGGTGACTGGCGGTTGTA
		R: GCCGTAGAGGTTGGCGTAG
*KLF7*	NM_001318990	F: GACACCGGCTACTTCTCAGC
		R: CTCGCACATACTCGTCTCCA
*SREBP1*	NM_204126	F: GGTCCGGGCCATGTTGA
		R: CAGGTTGGTGCGGGTGA
*FAS*	NM_205155	F: AAGGCGGAAGTCAACGG
		R: TTGATGGTGAGGAGTCG
*TBP*	NM_205103	F: GCGTTTTGCTGCTGTTATTATGAG
		R: TCCTTGCTGCCAGTCTGGAC
*A-FABP-ChIP*	NC_006089	F: ATGGAACAGGTGCTTCAACTCTCTC
		R: TTCACTTCTTTGGAGGGGAAATGAG
*LPL-ChIP*	NC_006127	F: CCCTTAAATAACTGATCCTTCACCC
		R: CGTACTGTGGAGAGACAGAGTTGCC

### RNA Interference

Synthetic small interfering (si) RNA oligonucleotides specific to the chicken *TCF21* mRNA regions were synthesized by GenePharma (Shanghai, China). The siRNA sequences was: 5′-GGAAATGCTGGAGTGCGAT-3′. The negative control siRNA sequence was: 5′-TTCTCCGAACGTGTCACGT-3′. After 36 h of differentiation, preadipocytes were transfected with these siRNA constructs using Lipofectamine RNAi MAX (Invitrogen) based on the manufacturer’s protocols. Cells were maintained in differentiation media until day 3 post-transfection.

### Plasmid Construction and Transfection

The full-length *TCF21* coding region was amplified via PCR using the following primers: *TCF21*-F (5′-CGGAATTCCCATGTCCACTGGGTCCCTCAGTG-3′) and *TCF21*-R (5′-GGGGTACCTCAGGATGCCGTCGGGC-3′), which were designed based on the chicken *TCF21* sequence (Accession No. NM_001277711.1). *Eco*RI and *Kpn*I recognition sites (underlined), respectively, were introduced. The PCR product was electrophoresed on a 1.5% agarose gel. The appropriate band was isolated, DNA was purified, and cloned into the *pEASY*^®^T1 Simple Cloning Vector (TransGen Biotech, Beijing, China). The cloned DNA sequence was verified by sequencing, and sequence analyses were performed using the DNAMAN v6.0 software (Lynnon Biosoft, Vaudreuil-Dorion, QC, Canada). *Eco*RI and *Kpn*I (Takara) were used to excise the *TCF21* coding region from the vector, which was then sub-cloned into the pCMV-HA vector (Clontech, Mountain View, CA, United States), yielding the final *TCF21* over-expression vector, termed pCMV-HA-*TCF21*.

Preadipocytes cultured in regular medium were transfected with either pCMV-HA-*TCF21* or an empty vector control (pCMV-HA) using Lipofectamine 2000 (Invitrogen) while at 70–90% confluency. Cells remained in regular medium for 24 h after transfection, followed by culture in differentiation media for another 3 days.

### Western Blotting

Protein was extracted from abdominal fat tissue and preadipocytes transfected with pCMV-HA-*TCF21* or pCMV-HA for 48, 72, and 96 h after lysis using RIPA buffer. Cellular extracts were supplemented with protease inhibitor cocktail, protein levels were measured and equal amounts were loaded on SDS-PAGE gels. After transfer to nitrocellulose membranes, blots were probed overnight at 4°C with the appropriate primary antibody (anti-TCF21, 1:50, Abmart; anti-HA, 1:1000, TransGen Biotech; anti-β-actin, 1:1000, TransGen Biotech) followed by an HRP-conjugated secondary antibody (1:5000, TransGen Biotech). Specific protein bands were visualized using the BeyoECL Plus kit (Beyotime) in a chemiluminescence system (Sagecreation, Beijing, China), and band intensity was quantified with the ImageJ program (NIH, Bethesda, MD, United States).

### Establishment of a Preadipocyte Cell Line Stably Over-Expressing TCF21

A lentivirus (LV) over-expression TCF21 (LV-TCF21) and a control LV (LV-control) were constructed by Hanbio (Shanghai, China). Immortalized chicken preadipocytes (ICP2) (Wang W. et al., 2017) were infected with LV-TCF21 or LV-control. Positive clones were selected based on blasticidin resistance and the presence of the ZsGreen gene in these constructs. Briefly, 72 h after infection, ICP2 cells were treated with 4 μg/ml blasticidin for 8 days. Then ZsGreen positive cells were sorted into a 96-well plate by flow cytometry, with one cell plated per well. Over-expression was confirmed by qPCR and Western blotting (Supplementary Material 1).

### Chromatin Immunoprecipitation

ChIP assay was performed on LV-TCF21 cells cultured in differentiation media for 24 h by SeqHealth (Wuhan, China). The cells were fixed in 1% formaldehyde for 10 min at room temperature, after which 0.125 M glycine was added for 5 min to terminate the crosslinking reaction. Cells were then collected, treated with nuclear lysis buffer, and sonicated to mediate DNA fragmentation. The sonicated chromatin was used in immunoprecipitation reactions with anti-HA antibody (Abcam) or with control rabbit IgG (Cell Signaling Technology). Specific primers were used to amplify the putative TCF21 binding sites within the promoter regions of A-FABP and LPL (Table 1). Data were presented as percentage of chromatin input.

### Luciferase Reporter Assay

To investigate how TCF21 influences A-FABP and LPL promoter activity, promoter regions from chicken *A-FABP* and *LPL* containing putative TCF21 binding sites (E-box motif: CAGCTG) were PCR amplified from chicken genomic DNA. Primer sequences were: A-FABP-F (5′-ATTTCTCTATCGATAGGTACCCCATGAAAGTTTTGGCACAGTTCTC-3′) and A-FABP-R (5′-CAGTACCGGAATGCCAAGCTTAGAAACGCTGCAGTGGCTCTAAAAT-3′); LPL-F (5′-ATTTCTCTATCGATAGGTACCACCGCATCAGTTGTCTTCT-3′) and LPL-R (5′-CAGTACCGGAATGCCAAGCTTTTTCCTGCGCTGTTGTAC-3′). KpnI and HindIII recognition sites (underlined) were introduced in these sequences. The resultant PCR products were purified and cloned into pGL3-basic luciferase reporter vectors (Promega, United States), forming constructs termed pGL3-basic-*A-FABP*-wild and pGL3-basic-*LPL*-wild. pGL3-basic-*A-FABP*-wild and pGL3-basic-*LPL*-wild constructs with mutated TCF21 binding sites were synthesized by GENEWIZ (Suzhou, China), and the resultant constructs were termed pGL3-basic-*A-FABP*-mutant and pGL3-basic-*LPL*-mutant.

These pGL3-basic-*A-FABP*-wild, pGL3-basic-*LPL*-wild, pGL3-basic-*A-FABP*-mutant, or pGL3-basic-*LPL*-mutant were co-transfected with either a mixture of pRL-TK Renilla luciferase vector (Promega, United States) and pCMV-HA, or a mixture of pRL-TK Renilla luciferase vector and pCMV-HA-TCF21 into a DF-1 or into ICP2 cells plated in 24 well plates. Cells were lysed 48 h post-transfection, and promoter activities were assessed with a Dual-Luciferase Assay System (Promega, United States) according to manufacturer’s instructions. Renilla luciferase activity was used to normalize promoter activity between samples.

### Statistical Analysis

All data are shown as means ± SD. Student’s *t*-tests were used to compare results between two groups. When more than two groups were compared, a generalized linear model (GLM) procedure followed by the Turkey’s HSD test was used according to the model: Y = μ + T +e, where Y represents the gene expression level, μ represents the population mean, T represents the fixed effect of time points during preadipocyte differentiation, and e represents the random residual effect. JMP v11.0 (SAS Institute, Inc., Cary, NC, United States) was used for all analyses, and the threshold of significance was *P* < 0.05 or *P* < 0.01.

## Results

### TCF21 Expression in the Abdominal Fat Tissues of Lean and Fat Line Chickens

**Figure 1:**
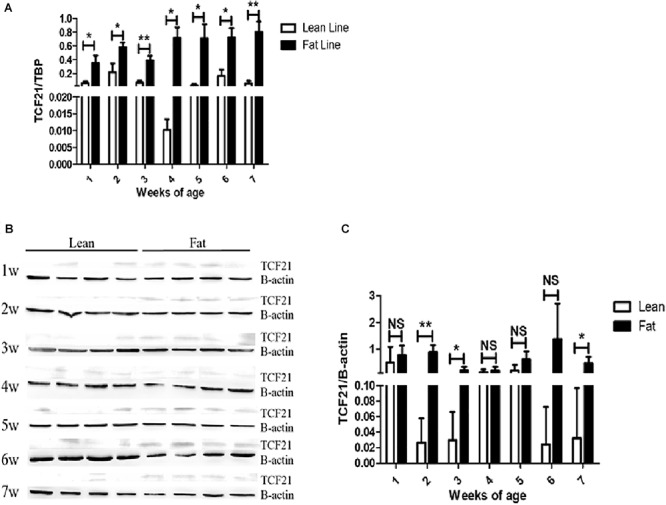
TCF21 expression in the abdominal fat tissues of lean and fat line chickens. **(A)** Relative *TCF21* mRNA levels in the abdominal fat of lean and fat broilers as measured by real-time quantitative PCR (*N* = 5 per line). **(B)** TCF21 protein levels in the abdominal fat of lean and fat broilers aged 1–7 weeks as measured by western blot (*N* = 4 per line). **(C)** Western blot data quantification (*N* = 4 per line). Data are means ± SD; ^∗^*P* < 0.05, ^∗∗^*P* < 0.01.

First, we analyzed TCF21 mRNA and protein expression in abdominal fat from lean and fat chickens ranging from 1 to 7 weeks of age. We found that *TCF21* mRNA expression was significantly higher in abdominal fat tissues of fat broilers relative to lean broilers at all seven time points (*P* < 0.05) (Figure 1A). Likewise, TCF21 protein levels were often elevated in fat broilers relative to lean broilers, and this difference was significant at 2, 3, and 7 weeks of age (*P* < 0.05) (Figure 1B,C).

### TCF21 Expression During Preadipocyte Differentiation

**Figure 2:**
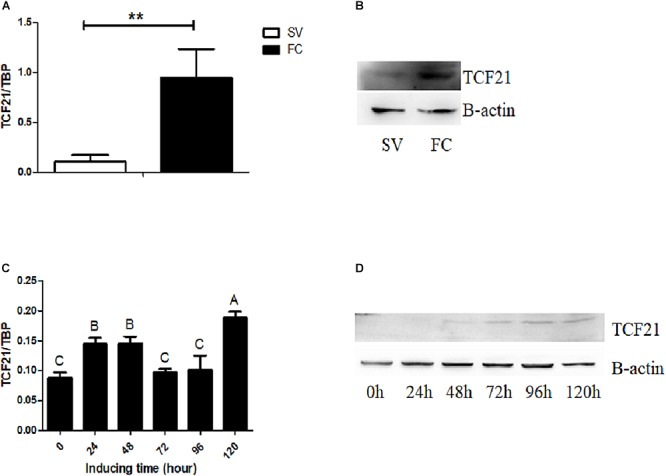
*TCF21* expression during preadipocyte differentiation. **(A)** *TCF21* expression in chicken preadipocytes (SV) and mature adipocytes (FC) as measured by qPCR (means ± SD; ^∗∗^*P* < 0.01). **(B)** Western blots analysis of TCF21 protein levels in chicken preadipocytes (SV) and mature adipocytes (FC). **(C)** *TCF21* mRNA expression pattern over the course of preadipocyte differentiation as measured by qPCR (means ± SD). Different uppercase letters above columns denote significant differences between time points. **(D)** Western blots analysis of TCF21 protein levels during preadipocyte differentiation. Experiments above were conducted thrice and in triplicates.

Next, we isolated chicken preadipocytes and mature adipocytes from the abdominal fat tissue of AA broilers, and used these cells to assess *TCF21* expression. We found that both TCF21 mRNA and protein levels were significantly higher in mature adipocytes than in preadipocytes (*P* < 0.01, Figure 2A,B). In addition, TCF21 mRNA increased at the early stage of differentiation, then dropped to the similar level to 0 h of induction, finally it significantly increased at 120 h after induction (Figure 2C), and TCF21 protein levels have an increasing trend (Figure 2D) during *in vitro* oleate-induced preadipocyte differentiation.

### Modulating TCF21 Expression Influences Preadipocyte Differentiation

Our results thus far suggested that *TCF21* may play a positive role in regulating chicken adipogenesis. To further test this hypothesis, we performed *TCF21* knock-down and over-expression experiments in preadipocytes. We assessed both lipid accumulation and the expressions of adipogenic genes [peroxisome proliferator-activated receptor gamma (PPARγ), CCAAT/enhancer binding protein alpha (C/EBPα), *A-FABP*, *LPL*, perilipin 1 (PLIN), kruppel like factor 7 (KLF7), sterol regulatory element binding protein 1 (SREBP1), and fatty acid synthase (FAS)] in order to investigate whether TCF21 knock-down or over-expression affects the preadipocyte differentiation.

**Figure 3:**
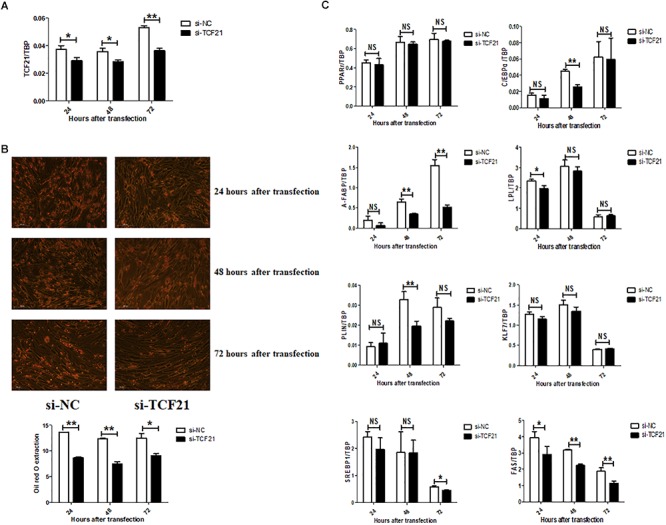
*TCF21* knock-down attenuates chicken preadipocyte differentiation. **(A)** *TCF21* mRNA knock-down efficiency in chicken preadipocytes as measured by qPCR after transfection with si-NC or si-TCF21 for 24, 48, and 72 h, respectively (means ± SD). **(B)** Oil red O staining of chicken preadipocytes transfected with si-NC or si-TCF21 (means ± SD). **(C)** Expression of adipogenic genes in chicken preadipocytes measured by qPCR after transfection with si-NC or si-TCF21 (means ± SD). ^∗^*P* < 0.05, ^∗∗^*P* < 0.01. Experiments above were conducted thrice and in triplicates. PPARγ, peroxisome proliferator-activated receptor gamma; C/EBPα, CCAAT/enhancer binding protein alpha; A-FABP, adipocyte fatty acid binding protein; LPL, lipoprotein lipase; PLIN, perilipin protein; KLF7, kruppel like factor 7; SREBP1, sterol regulatory element binding transcription factor 1; FAS, fatty acid synthase.

**Figure 4:**
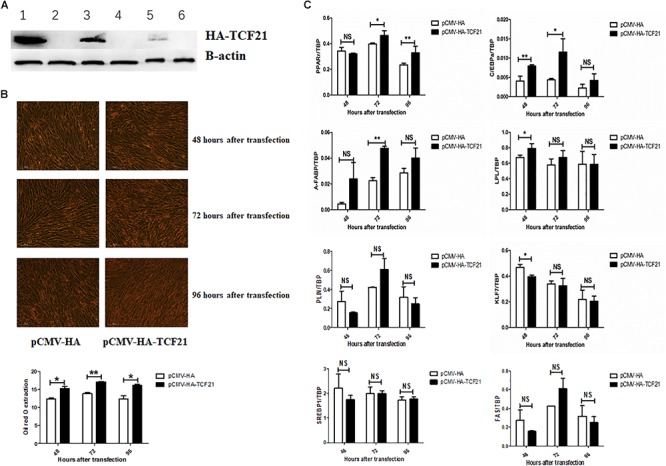
*TCF21* over-expression promotes chicken preadipocyte differentiation. **(A)** TCF21 protein over-expression efficiency in chicken preadipocytes as measured by western blot after transfection using pCMV-HA or pCMV-HA-TCF21. Lanes 1, 3, 5 and lanes 2, 4, 6 were chicken preadipocytes transfected with pCMV-HA-TCF21 and pCMV-HA, respectively, for 48, 72, and 96 h. **(B)** Oil red O staining of preadipocytes transfected using pCMV-HA or pCMV-HA-TCF21 (means ± SD). **(C)** Expression analysis of adipogenic genes in chicken preadipocytes measured by qPCR after transfection with pCMV-HA or pCMV-HA-TCF21, respectively (means ± SD). ^∗^*P* < 0.05, ^∗∗^*P* < 0.01. Experiments above were conducted thrice and in triplicates. PPARγ, peroxisome proliferator-activated receptor gamma; C/EBPα, CCAAT/enhancer binding protein alpha; A-FABP, adipocyte fatty acid binding protein; LPL, lipoprotein lipase; PLIN, perilipin protein; KLF7, kruppel like factor 7; SREBP1, sterol regulatory element binding transcription factor 1; FAS, fatty acid synthase.

Lipid accumulation significantly decreased after knock-down of TCF21 (Figure 3A), as evidenced by oil red O staining (Figure 3B). In addition, the expression levels of pro-adipogenic genes *C/EBPα* (48 h after transfection), *A-FABP* (48 and 72 h), *LPL* (24 h), *PLIN* (48 h), *SREBP1* (72 h), and *FAS* (24, 48, and 72 h) were markedly down-regulated (Figure 3C). Over-expression of *TCF21* (Figure 4A) led to significant increases in intracellular lipid accumulation in chicken preadipocytes at all three time points (48, 72, 96 h) (Figure 4B). Consistent with this finding, the expression levels of pro-adipogenic genes including *PPARγ* (72 and 96 h), *C/EBPα* (48 and 72 h), *A-FABP* (72 h), and *LPL* (48 h) was significantly elevated (*P* < 0.05 or *P* < 0.01), whereas the expression level of the anti-adipogenic gene *KLF7* (48 h) was significantly decreased (*P* < 0.05, Figure 4C).

### TCF21 Promotes the Expression of LPL by Directly Binding to Its Promoter Region

**Figure 5:**
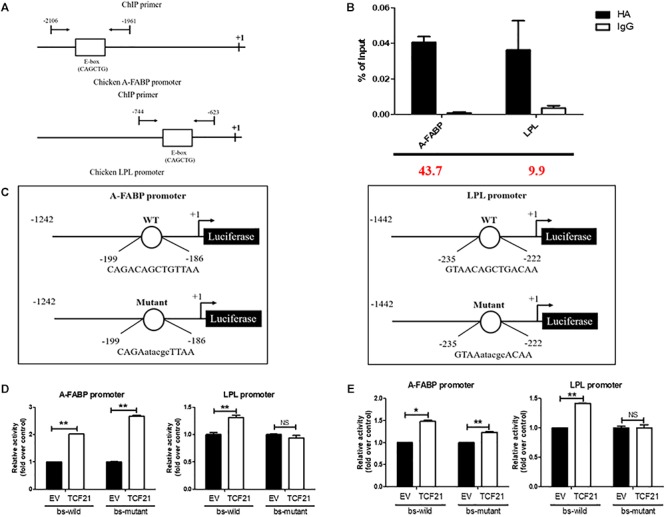
LPL is a target of TCF21. **(A)** Schematic diagram of the chicken *A-FABP* and *LPL* promoter sequences, with arrows indicating the forward and reverse primers used to amplify ChIP products. **(B)** Verification of chromatin immunoprecipitation by qPCR. Three independent immunoprecipitation reactions were analyzed, and the fold enrichment is provided in red at the bottom of the image. **(C)** Schematic representation of the *A-FABP* and *LPL* promoter constructs used in luciferase-based experiments, with letters indicating TCF21 binding site mutations. **(D)** Luciferase activity assay in DF-1 cells after co-transfection of pGL3-basic-*A-FABP* or pGL3-basic-*LPL* with either wild-type or mutated TCF21 binding site (bs) and the pCMV-HA or pCMV-HA-*TCF21* vector (means ± SD). ^∗∗^*P* < 0.01. **(E)** Luciferase activity assay in ICP2 after co-transfection of pGL3-basic-*A-FABP* or pGL3-basic-*LPL* with either wild-type or mutated TCF21 binding site (bs) and the pCMV-HA or pCMV-HA-*TCF21* vector (means ± SD). ^∗^*P* < 0.05, ^∗∗^*P* < 0.01. Experiments conducted thrice and in triplicates for results presented in **(B,D,E)**. A-FABP, adipocyte fatty acid binding protein; LPL, lipoprotein lipase; TCF21, transcription factor 21.

The evidence provided above supports that TCF21 promotes the differentiation of chicken preadipocytes. Further bioinformatic analyses predicted potential TCF21 binding sites within the promoters of both *A-FABP* (-2052 to -2038 bp from the transcriptional start site; Supplementary Material 2) and *LPL* (-702 to -688 bp from the transcriptional start site; Supplementary Material 3). To confirm that TCF21 could physically bind to these promoters, we performed ChIP in LV-TCF21 preadipocytes 24 h after the initiation of differentiation. ChIP-qPCR revealed that TCF21 could bind the E-box motif (CAGCTG) present in the *A-FABP* and *LPL* promoters (Figure 5A,B). To further confirm whether TCF21 binding was able to directly regulate the transcriptional activities of *A-FABP* and *LPL*, we cloned *A-FABP* and *LPL* promoter sequences harboring wild-type or mutated E-box motifs into the pGL3-basic vector up-stream of a luciferase reporter gene (Figure 5C). Luciferase reporter assays of these constructs revealed that TCF21 over-expression in DF-1 cells significantly induced transcription from both promoters when the E-box sequence was present (Figure 5D). Furthermore, when the binding site within the *LPL* promoter was mutated, this TCF21-dependent induction of gene expression was lost. However, no change was observed in *A-FABP* if the putative binding site was mutated (Figure 5D). This finding was further confirmed in ICP2 cells (Figure 5E). Together, these data indicate that *LPL* is a *bona fide* TCF21 target gene involved in preadipocyte differentiation.

## Discussion

Excessive fat deposition in chickens can result in many undesirable consequences within the poultry industry, including reduced feeding efficiency (Zhou et al., 2006) and decreased reproductive performance (Walzem and Chen, 2014). Thus, broiler breeders urgently require a more thorough understanding of the molecular mechanism governing chicken adipogenesis. We previously found that broilers from lean and fat lines exhibit clear differences in reproductive performance (Zhang et al., 2018). In addition, TCF21 was found to be related to testis growth and development in these broilers (Zhang et al., 2017). In mice and humans, TCF21 has been reported to not only influence reproduction (Bhandari et al., 2012), but to also regulate the development of white adipose tissue (Timmoms et al., 2007; Petrovic et al., 2010). Therefore, we sought to investigate whether TCF21 plays a similar role in regulating chicken preadipocyte differentiation.

First, we first compared TCF21 expression in the adipose tissue of lean and fat chickens from 1 to 7 weeks of age. We found that, similar to prior findings in the visceral adipose tissue of normal and obese Uygurs (Zhang et al., 2015), both TCF21 mRNA and protein expression levels were significantly higher in the abdominal adipose tissue of fat relative to lean birds. In addition, TCF21 mRNA and protein levels were markedly higher in mature chicken adipocytes relative to preadipocytes *in vivo*, and TCF21 expression had an increasing trend over the course of preadipocyte differentiation *in vitro*. These data suggest that TCF21 is involved in chicken adipose tissue growth and development, and indicate that TCF21 may play a role in preadipocyte differentiation.

Next, we next conducted knock-down and over-expression experiments to more closely examine the effect of TCF21 on preadipocyte differentiation. Accumulation of lipid droplets was decreased in TCF21 knock-down cells and increased in TCF21 over-expressing cells, demonstrating that TCF21 promotes preadipocyte differentiation. Preadipocyte differentiation is a complex process orchestrated by many genes. Therefore, we measured the expressions of genes known to play key roles in preadipocyte differentiation and lipid metabolism, including *PPARγ*, *C/EBPα*, *A-FABP*, *LPL*, *PLIN*, *KLF7*, *SREBP1*, and *FAS* (Cryer, 1981; Amri et al., 1991; Rosen et al., 2000; Linhart et al., 2001; Kawamura et al., 2006; Park et al., 2018). TCF21 knock-down reduced the expressions of pro-adipogenesis genes (*C/EBPα*, *A-FABP*, *LPL*, *PLIN*, *SREBP1*, and *FAS*), while TCF21 over-expression increased the expressions of pro-adipogenesis genes (*PPARγ*, *C/EBPα*, *A-FABP*, *LPL*) and decreased anti-adipogenic *KLF7* expression. Thus, TCF21 functions in promoting preadipocyte differentiation. Of note, we observed that the expression levels of *C/EBPα*, *A-FABP*, *LPL* were respectively up- or down-regulated upon TCF21 over-expression and knock-down, respectively, which suggested that these three genes are regulated by TCF21 and may play a role in the mechanism by which TCF21 regulates adipocyte differentiation.

Predictive bioinformatics analysis indicated that a potential TCF21 binding site (CAGCTG) was present in the *A-FABP* and *LPL* promoters. A subsequent chromatin immunoprecipitation experiment revealed that TCF21 directly binds to the promoters of both *A-FABP* and *LPL*. Luciferase reporter assays using these promoter sequences showed that *LPL*, but not *A-FABP*, was a direct target of TCF21 in the context of preadipocyte differentiation. The role of LPL in adipogenesis has been previously studied, and expression of LPL messenger RNA was observed in the early stages of adipogenesis and it reached a stable level in mature adipocytes, which indicates LPL is one of the critical factors in the process of adipogenic differentiation (Kim et al., 2014). In addition, Hu et al. (2018) certified that LPL promoted adipogenic differentiation. LPL also plays an important role in lipid metabolism that LPL catalyzes the hydrolysis of lipoprotein-derived triacylglycerol and allows the accumulation of fatty acids in adipose tissue, which are re-esterified in adipocyte to form stored triacylglycerol (Cryer, 1981; Wang G. et al., 2017). Therefore, TCF21 could bind directly to the *LPL* promoter and drive *LPL* expression to promote preadipocyte differentiation and lipid accumulation. The observation that *A-FABP* was not directly regulated may be explained by the influence of other transcription factors, which may bind to the promoter of *A-FABP* and form a complex with TCF21. Together, these data allow us to conclude that TCF21 promotes preadipocyte differentiation through two possible mechanisms: TCF21 binds directly to the *LPL* promoter and drives *LPL* expression, and TCF21 may indirectly promote the expression of *A-FABP*.

In summary, this study was the first to show that TCF21 is involved in adipose growth and development in chickens through the promotion of preadipocyte differentiation via targeting *LPL*. Thus, our findings further the overall understanding of adipogenic regulation in chickens, providing a foundation for future research into the underlying molecular mechanisms governing this process and how TCF21 promotes preadipocyte differentiation in these animals.

## Author Contributions

HL conceived this study. HL, BC, and XZ designed the manuscript. XZ performed the experiments, analyzed the data, wrote the manuscript. HL, YL and ZC contributed the reagents, materials, and analysis tools. HL, CL, ZD, HZ, NW, and MW helped to revising this manuscript.

## Conflict of Interest Statement

The authors declare that the research was conducted in the absence of any commercial or financial relationships that could be construed as a potential conflict of interest.

## Abbreviations

AA: Arbor Acres
A-FABP: adipocyte fatty acid binding protein
AFP: abdominal fat percentage
BW7: body weight at 7 weeks of age
bHLH: basic helix-loop-helix
C/EBPα: CCAAT/enhancer binding protein alpha
DF1: chicken embryo fibroblast cell line
DMEM: Dulbecco’s modified Eagle’s medium
FAS: fatty acid synthase
G: generation
KLF7: kruppel like factor 7
LPL: lipoprotein lipase
NEAUHLF: Northeast Agriculture University broiler lines divergently selected for abdominal fat content
PBS: phosphate buffer saline
PLIN: perilipin 1
PPARγ: peroxisome proliferator-activated receptor gamma
RIPA: radio immunoprecipitation assay
si: small interfering
SREBP1: sterol regulatory element binding protein 1
TBP: TATA-box binding protein
TCF21: transcription factor 21
TeW: testis weight.
