# Supplementary material for: A Novel Regulator of Preadipocyte Differentiation, Transcription Factor TCF21, Functions Partially Through Promoting LPL Expression
Source: Front Physiol. 2019 Apr 23;10:458. doi: 10.3389/fphys.2019.00458 (PMC6489524; doi:10.3389/fphys.2019.00458)
Supplement: Supplementary file 1 [file Presentation_1.pdf]

Supplementary material 1. The identification of stable cell line over-expressing TCF21 by real-time quantitative PCR and western blot.

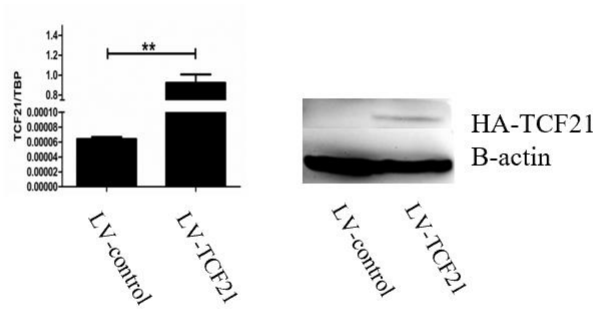

Supplementary material 2. Prediction of TCF21 binding site on the chicken A-FABP promoter. The predicted binding site sequence of TCF21 was marked in red. Partial 5’-untranslated region were capitalized and marked in blue.

caagaaatttacaaatagctcagattgacttcaaataggtgcaagcaattgattgcctggggtgagtgagcttgtctcctccaa
acagatttacatggaacaggtgcttcaactctctcccaaaaaccattttccagcaaaagctttgcagacagctgttaacatgga
aggaaagccaattcccactttcaggttgtgtttttgaatataaaagcctcatttcccctccaaagaagtgaatttagtaagtcac
acacagaatagttcccaaaagacaagaaaagtaagtgcaggaactgagttcaacaagtttacatcctttattttagagccact
gcagcgtttctcaccttatactttcaagtatttatatttttactggatttgagttttcaggtttctacagacttctcacacatagtgtag
tgtttctcagtccaataatcctcagtaataaaaagacacaatcttcaggtccttcagaatttccttctaaagagaatctgagcatg
tgatacactgagaaagccaatcagtagagcaattgggggcttccataattagaatattattttatttgttttaggattaacccaaa
gtcaataaatacacaaagactcaaaaaaagaacgggccaaacttcagatttagatattctcgggggaaaatgagatggagtt
aaaaaaatctttttgttgtttttctcaaacagagcctgcagtctaaaagggacaaaagggtaactaatcatacacctacagga
atattcaggttgtgcagcaatgaaatggggatgaaccggcaggggagcagctcagcagccctaaaggtggcaacagaaa
ggccatggatgcttacaacgtaacatctatggaatgcgacagacgagtagatgagttggaaccaaggataaaagccaaag
gatagaagttaaagaggagcatttcagactggagatgaggcaactgtttccccagggacacagggctggacagggagcg
agtttcccacgaggctgtgcaactccatcctcagagttgtccaagtttccccagaggatgcagccctgggcagcccggtgt
gagctggccctgcccaaggctgcaccatcctgtctgaactttcctataatcttacaatcctatacaaataatttttatataacattg
ttatatcaaaatatgtggaccaacacatttgtttcagtgatcagaaaagtatgttgaagaaatccacctaaagaagaatgcata
ggattgaatgcttgtgtttttctgaccatgtaatagcatgttacagccagaaggaacatctagtaaattgcttgcttaatgcctc
agttcttcatgatctgagctgagagtagaccttgttatgctaggcctgtatacattttgtctcgagctgataattacataaatagta
caaacagaaagaaaatgataaatccagagaccttcagtggactgataccagtacctagatttttgcaataagtaatcacaatg
ctagtcattgggacacaggtctccagagggaacacaagtcaaagtgggacaagtcatcaactctgttactctgcatttcatag
aacgctgtgtccccactgcatcccaggaaaggagaatacagacctattggatctcagtcctcagccaaggaagtaggatat
atccttctacagccaccggcactcccactttctctccccacatccactttttcctctagttctgcgcctctctgtagaaaatcacta
actgtagttgtgcaaaaagaaaattaaatgatcccaagtctttcagatcttgcagtctaggaatttagctgttttcatacaatatg
gaggactgaaatgaaagaaaaggaatataggagacagcataccctctggggggttgaacctaactttttattctcaaagaaaa
tattattttaatatttgcttttatggtgaagtgtcctcttgaattatgaaaataattaaataaaaaaatgacagtgatctttgtgctagt
aaaggcatatgaataataacactgtgacctactggcaaaggaggggtaggggaggaaggaaaacactgggcactaagg
actccctctttattggtcatcctagtacatcatgtggccaaggctcctttttaaagcccatctcttggagctcatactatcctcaca
gcttctgtttctgcttGATCCTGTGAAAGACTGCTACCTGGCCTGACAAA

Supplementary material 3. Prediction of TCF21 binding site on the chicken LPL promoter. The predicted binding site sequence of TCF21 was marked in red. Partial 5’-untranslated region were capitalized and marked in blue.

ccctgcacccagctcacgttggctggaagaaagactcagtctcagctgcaccttctttaaattcttccattgccttttgggtatg
aaaagctgcatcccaggagacatcacagaatcgtagaatcattaaggctggaaatgacctctaagaccgccaaatcccca
ccacgaccactaaccacgtccctcagtgccacatccacctggttcttgaatacctccacagacagtgacttcatcacctccac
gggcagcctgtgccactgcttcagcactctttcagagaagtttttctccacacccaatctgagatgcatacttgcgctgggatc
tcactggtcctctatcagatcggcaccttgcagaggtgacccagtgctgctctgcctgcaccaacgcctgtgcctatgggga
ccaggagtgttttaattcccactctgagaaaccactgcccttgaagtgaatggcgtttgctccatgctgccagaaccaagga
gccctccttctctacaccaaaagcacctcgtgaagccaagcctttgacaaaacaatgcacagtaaataaaagcaaggctca
ctattaatttaatatttctctgttacattcccttaaataactgatccttcaccctgtggcagcagcaaaaggtaacagctgacaaa
ctgctggcagaggggcacagagaggtgaaaaactaggagaggcaactctgtctctccacagtacgcacggcagcctcca
gcaacttgcactccgagctcgcaccccccttggttgttggcaaaacaaagcattggcaaaggcatttcacgcgagcagcaaa
aggtgcttcgcaagcagcgagctgagcaccagtcgcttcttggtacaacagcgcaggaaagagggaagcttagcgttcg
aggctcggggagccaggacggcgaacacaggggaagggccgtgcaggagggctgcgaggctcagctcagcgggac
gccgagggggcaaagagcgggggcagcgcgcccagtccgagggctcagagccggcgacggagccgcacccgctgg
cggagctcgcggctcccagccgtgcccggggccgtgcgggggcgcgagggtcgcggggccccgcgggagcggccc
ggccctcccccccccaccgcctgtgcccgggccagcggtgaccacgccccgtggcgtgcgccaatgggtgtgggggctc
atttgcatatttgcatattggcggggacttaaggggccgggagggagcggcggcagtggtttgcctataggtttcggaggg
aggggtcgtcgctgcgggtttctcccggagcgactcagttctacttcgtgagtttttttttttttCCTTCACAGTCGT
GTGTTTTAGAACTTAGTTATTCTATTTTGTTTTGTTT
